# Effect of scaffold elasticity on the gene expression of annulus fibrosus-derived stem cells

**DOI:** 10.1016/j.dib.2015.11.028

**Published:** 2015-11-21

**Authors:** Caihong Zhu, Jun Li, Chen Liu, Pinghui Zhou, Huilin Yang, Bin Li

**Affiliations:** Department of Orthopaedics, The First Affiliated Hospital, Orthopaedic Institute, Soochow University, 188 Shizi St, Suzhou, Jiangsu 215006, China

**Keywords:** Elasticity, Annulus fibrosus-derived stem cells, Gene expression

## Abstract

This article provides more experimental details and findings of the study as to how the elasticity of scaffold material modulates the gene expression of annulus fibrosus-derived stem cells (AFSCs) (Zhu et al., 2015 [1]). The detailed synthetic route and characterizations of four kinds of biodegradable poly(ether carbonate urethane)ureas (PECUUs) are described. After AFSCs were cultured on electrospun PECUU fibrous scaffolds, the cell proliferation and gene expression analyses were performed to explore the effect of substrate elasticity on the growth and differentiation characteristics of AFSCs.

**Specifications Table**

**Table:** 

Subject area	Materials, biology, bioengineering
More specific subject area	Biomaterials and tissue engineering
Type of data	Scheme, figure, table
How data was acquired	Differential scanning calorimetry (DSC), tensile tests, nanoindentation, immunofluorescence, real-time quantitative PCR (RT-qPCR)
Data format	Raw and analyzed data
Experimental factors	PECUUs scaffolds with different elasticity
Experimental features	Synthesis and mechanical and calorimetric measurements of polymers, cell culture tests followed by gene analysis
Data source location	Suzhou, China
Data accessibility	Data is provided in the article

**Value of the data**

1. Demonstrate that strong PECUUs with different elasticity can be readily synthesized by adjusting the molecular composition.
2. Confirm that the elasticity of substrate material markedly affects the gene expression of AFSCs on electrospun scaffolds.
3. Compare side-by-side the elasticity of PECUUs to that of various regions of native AF tissue.

## 1 Data

### 1.1 Synthesis of PECUUs with tunable elasticity

**Scheme 1:**
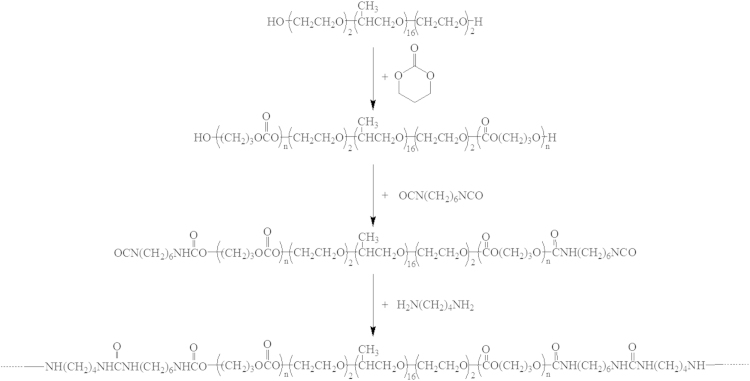
The synthetic route of PECUUs.

The PECUUs were synthesized through a one pot, three-step polymerization process (Scheme 1) [1], [2]. By changing the ratios of PEO-PEO-PPO/TMC, three kinds of polydiols were synthesized, which are named PTMC_0_-PEO-PPO-PEO-PTMC_0_, PTMC_10_-PEO-PPO-PEO-PTMC_10_ and PTMC_17_-PEO-PPO-PEO-PTMC_17_, respectively. After that, four kinds of PECUUs (PECUU-1, PECUU-2, PECUU-3, and PECUU-4, respectively) were prepared by changing the ratios of soft segment to hard segment, i.e., the ratios of putrescine/1,6-diisocyanatohexane/polydiols.

### 1.2 Mechanical properties of PECUUs

**Fig. 1:**
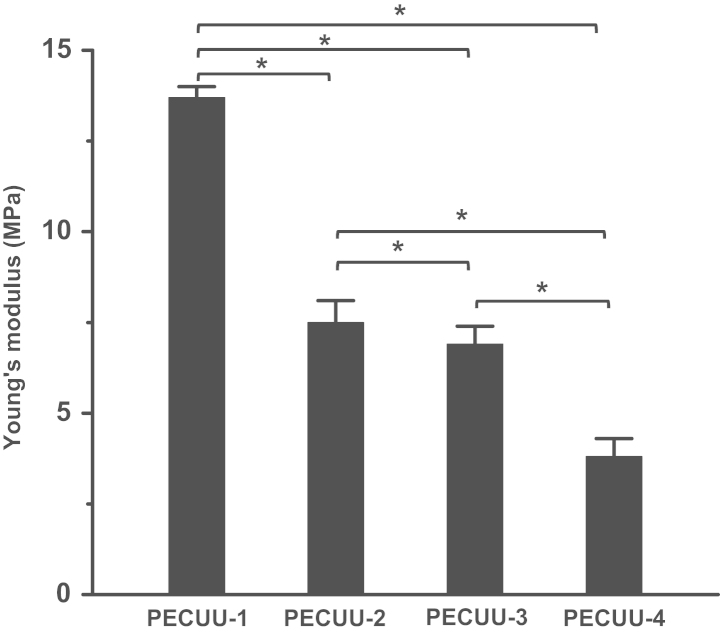
The Young׳s moduli of PECUUs measured using nanoindentation tests. Asterisk(*) indicates significant difference between groups (*p*<0.05, *n*≥10).

The elastic moduli of PECUUs, measured using nanoindentation, was 13.4±1.7, 6.4±0.5, 5.1±0.2, and 2.5±0.2 MPa for PECUU-1, PECUU-2, PECUU-3, and PECUU-4, respectively (Fig. 1). In addition, the mechanical properties of PECUU fibrous scaffolds fabricated by electrospinning technique were measured using uniaxial tensile tests (Table 1).

### 1.3 Differential scanning calorimetry (DSC) analysis of PECUUs

**Fig. 2:**
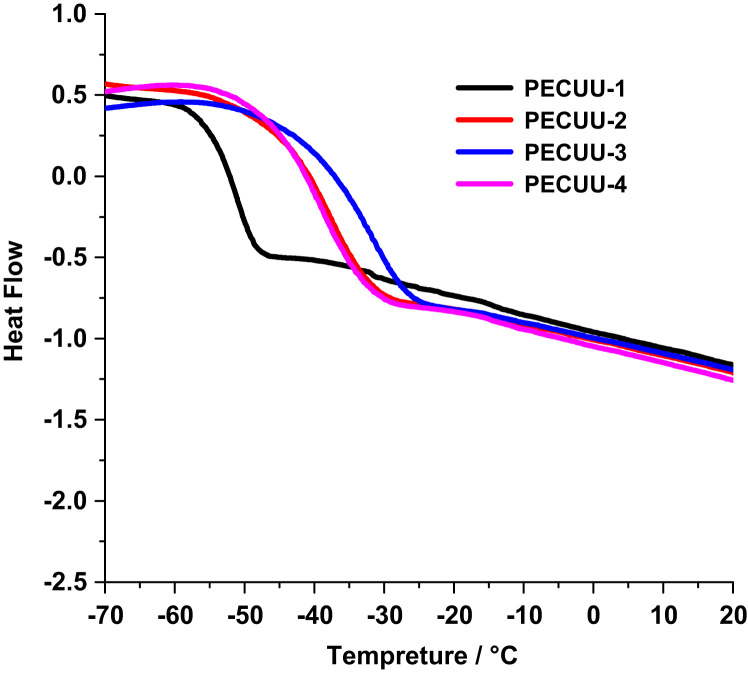
DSC curves of PECUUs. Heating rate, 20 °C min^−1^.

All the PECUUs had glass transition temperatures lower than −37 °C (Fig. 2). The glass transition temperature of PECUU increased from −52 °C (PECUU-1) to −37 °C (PECUU-3) when the PTMC length was increased from 0 g/mol (PTMC_0_-PEO-PPO-PEO-PTMC_0_) to 3460 g/mol (PTMC_17_-PEO-PPO-PEO-PTMC_17_). When the PTMC length was similar, the glass transition temperatures of PECUUs remained to be −41 °C for both PECUU-2 (ratio: 2/2/1) and PECUU-4 (ratio: 1.5/1.5/1) even though the feed ratios of hard segment to soft segment (putrescine/1,6-diisocyanatohexane/polydiols) were different.

### 1.4 Mechanical properties of PECUUs after in vitro degradation

After the cast PECUU membranes were incubated in PBS for 1, 2, 3, and 4 weeks, their mechanical properties were measured using uniaxial tensile tests (Table 2).

### 1.5 Immunofluorescence of AFSCs cultured on PECUUs

**Fig. 3:**
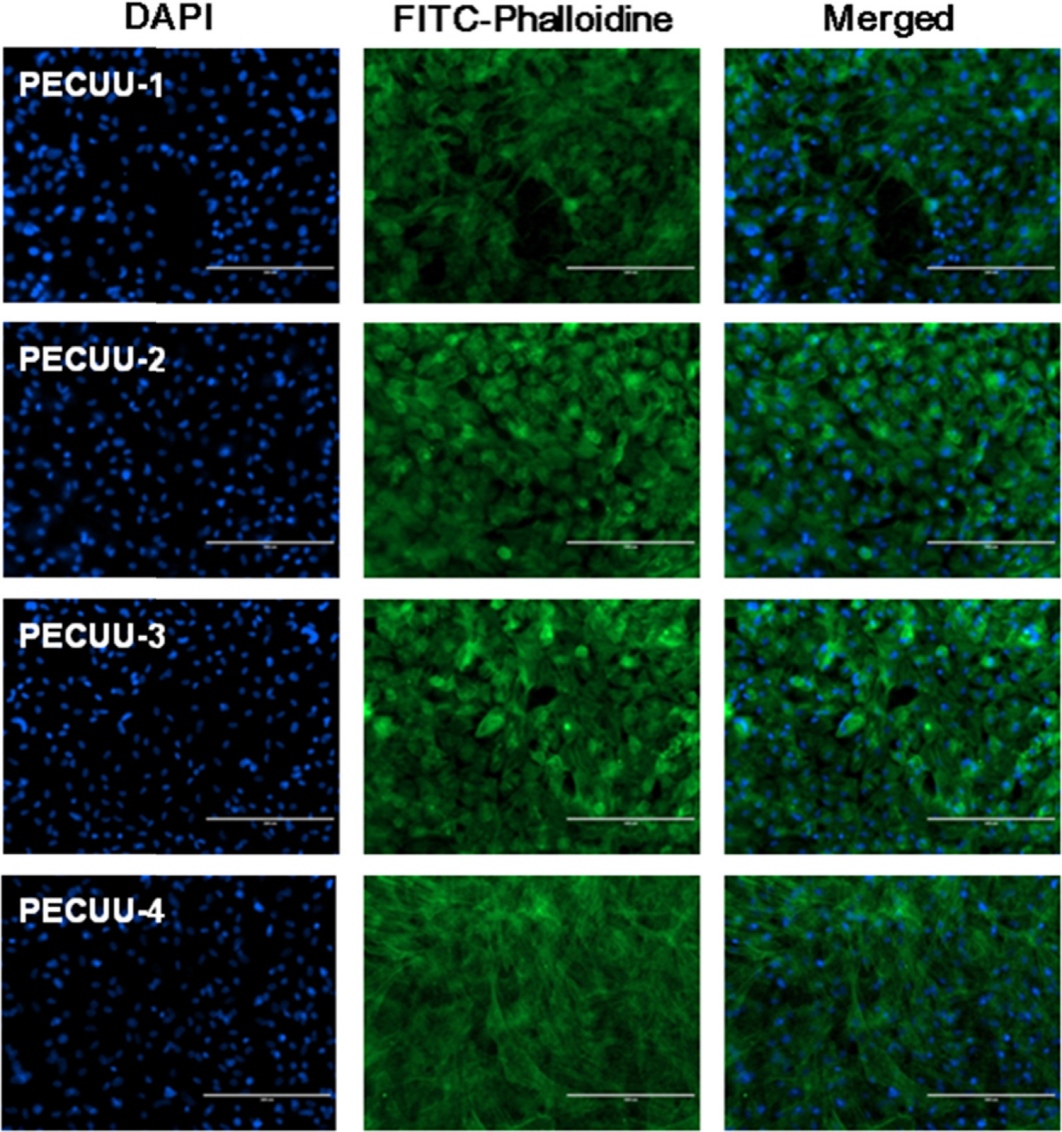
Immunofluorescence images of AFSCs cultured on PECUUs for 2 weeks.

The morphology of AFSCs cultured on PECUUs for 2 weeks is shown in Fig. 3. The immunofluorescence pictures of F-actin and nuclei indicate that AFSCs spread and grew well on all the electrospun PECUU fibrous scaffolds.

### 1.6 Gene expression of AFSCs cultured on PECUUs for 2 weeks

**Fig. 4:**
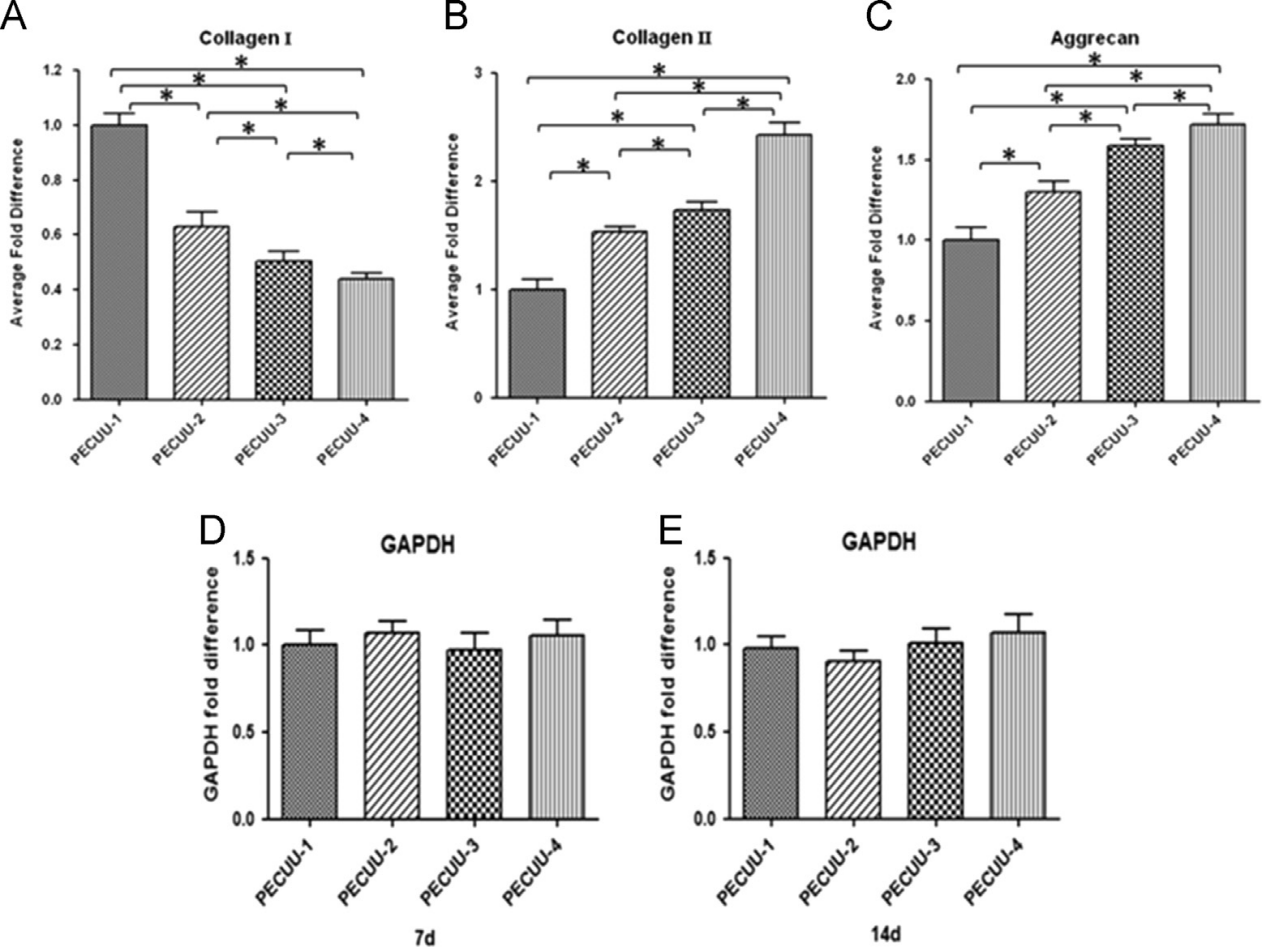
Real time quantitative PCR analyses for the expression of collagen-I, collagen-II, and aggrecan genes, respectively, in AFSCs cultured on PECUU scaffolds for 2 weeks (A–C). Gene expression was normalized to GAPDH expression. GAPDH expression remains stable in all culture conditions (D, E).

The gene expression of major matrix components of AF tissue, including collagen-I, collagen-II and aggrecan, after 2 weeks of culture was examined in AFSCs cultured on electrospun PECUU scaffolds using qRT-PCR analysis. The expression of collagen-I gene in AFSCs appeared to increasing with the increase of PECUU elasticity after being cultured for 2 weeks (Fig. 4). For example, the expression of collagen-I in AFSCs cultured on PECUU-1 (*E*=13.4 MPa) was nearly 2 times higher than that of cells on PECUU-4 (*E*=2.5 MPa). In contrast, the gene expression of collagen-II and aggrecan decreased with the increase of substrate elasticity (Fig. 4). The expression levels of collagen-II and aggrecan in AFSCs on PECUU-4 were 2.4 and 1.7 times, respectively, higher than those of cells on PECUU-1.

### 1.7 The cellular biochemistry and mechanics of AFSCs cultured on PECUUs

The biochemical measurements of major AF matrix components in AFSCs cultured on various electrospun PECUU scaffolds were performed after 2 weeks (Table 3). The cell traction forces were measured as well. For convenience of comparison, the corresponding characteristics of different regions of native AF tissue are also provided side by side.

## 2 Experimental design, materials and methods

### 2.1 Synthesis of PECUUs with tunable elasticity

The PECUUs were synthesized by a one pot, three-step polymerization process as shown in Scheme 1. In brief, the ring-opening polymerization of trimethylcarbonate (TMC) was carried out at 110 °C for 24 h with PEO-PPO-PEO as the initiator and stannous octoate as the catalyst. The resulting polydiols were used as soft segment and reacted with 1,6-diisocyanatohexane (HDI) at 75 °C for 4 h under continuous stirring in 15 wt% toluene. Following that, 1 wt% putrescine in DMF was added dropwise as a chain extender to obtain PECUU.

### 2.2 Measurement of the elastic moduli of PECUUs using nanoindentation

The membranes of all four kinds of PECUUs were prepared using solution cast method. Then the modulus of membranes was tested with a nanoindentation system (Nano Indenter G200, Agilent). As previously described [3], dynamic indentations with five running frequencies (10, 5.62, 3.16, 1.78 and 1 Hz) and 50 nm oscillation amplitude were performed using the “G-Series XP CSM flat punch complex modulus’’ module of the NanoSuite method at the axial direction using a flat indenter(diameter, 215 mm) at approximately 22 °C. At least ten indentations were measured for each sample.

### 2.3 DSC analysis of PECUUs

DSC analysis was performed on a DSC instrument (Perkin-Elmer) within the range of −80–150 °C at a heating rate of 20 °C min^−1^.

### 2.4 AF tissue harvest for isolating AFSCs

**Fig. 5:**
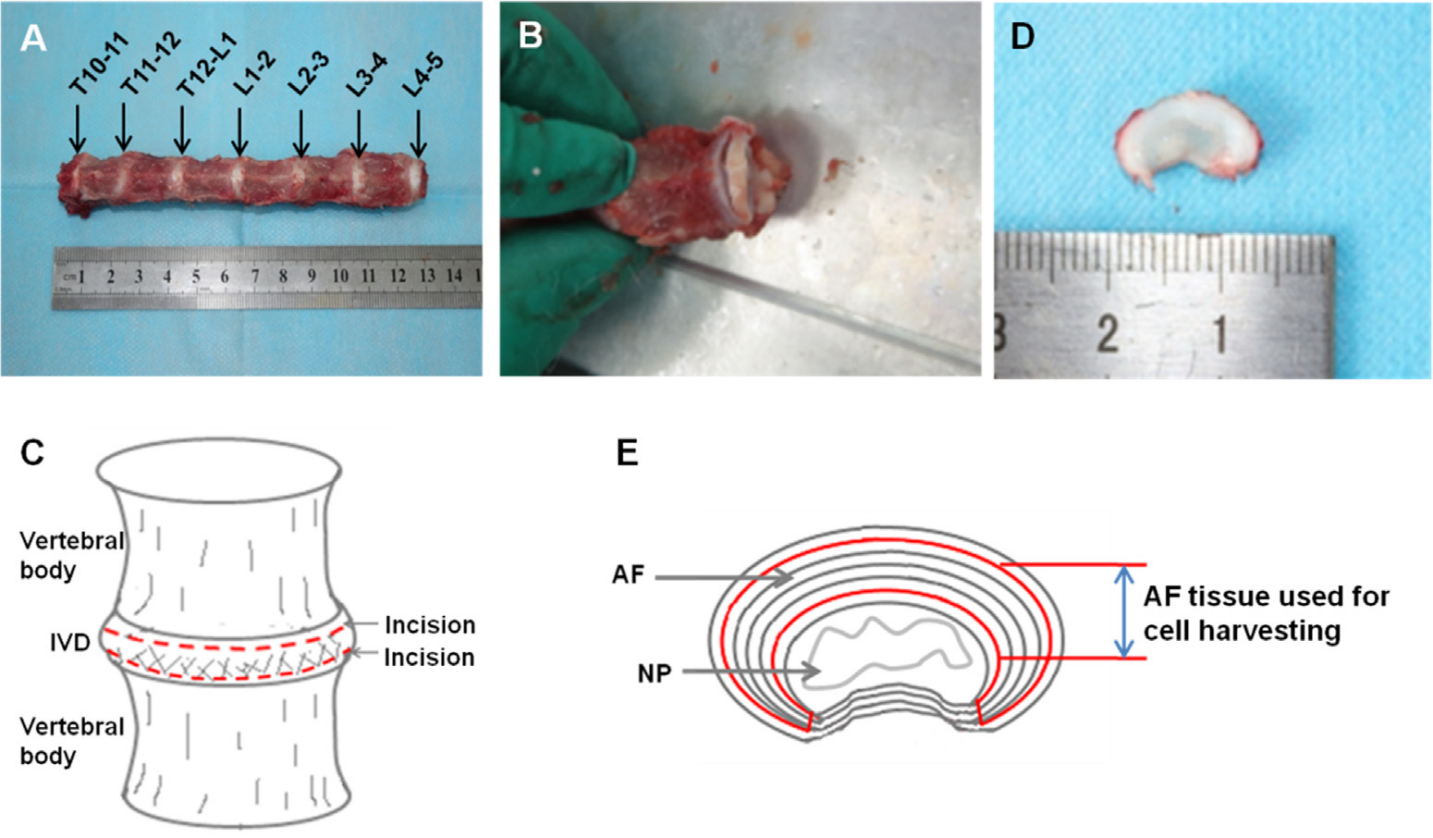
Harvest of AF tissues for isolating AFSCs.

After the muscles and ligaments were removed, IVDs from T10 to L5 were isolated (Fig. 5). The spinal column was then transversally sectioned around the middle of each disc. After a completed disc was harvested, pure AF tissue was obtained by carefully removing the NP, inner and outer AF tissues.

### 2.5 Gene expression of AFSCs on electrospun PECUU scaffolds

The electrospun scaffolds of PECUUs were fabricated as previously described [4]. The AFSCs were isolated as previously described and used at passage 1 [5]. PECUU substrates of different elasticity were cut to fit into a 96-well plate for cell morphology observation and into a 24-well plate for gene expression tests, respectively. For cell morphology test, AFSCs were seeded at the density of 2000 cells/well in 96-well plate and then cultured in low glucose DMEM supplemented with 10% FBS. After 2 weeks, the morphology of cells was evaluated by sequentially staining with DAPI and phalloidin-FITC for visualizing F-actin and nuclei, respectively. For gene expression analysis, AFSCs were seeded at the density of 5×10^4^ cells/well and then cultured in low glucose DMEM supplemented with 10% FBS. After 2 weeks, total RNA were extracted using TRIZOL isolation system (Invitrogen) and analyzed by real-time quantitative PCR (RT-qPCR) (CFX96, Bio-Rad) using the SsoFast EvaGreen supermix kit (Bio-Rad). The primers for GAPDH, collagen-I, collagen-II and aggrecan were designed using the mRNA sequences deposited in Gene Bank as mentioned in our previously published papers [3], [4], [6].

## Figures and Tables

**Table 1:** Mechanical properties of electrospun PECUU scaffolds tested by uniaxial tensile tests at different conditions (sample thickness, ~0.1 mm; *n*=4).

**Sample**	**Young׳s modulus (MPa)**		**Tensile strength (MPa)**		**Elongation at break (%)**	
**22 °C, air**	**37 °C, water**	**22 °C, air**	**37 °C, water**	**22 °C, air**	**37 °C, water**
PECUU-1	5.0±0.5	0.4±0.0	9.2±0.4	1.0±0.3	240±10	207±38
PECUU-2	1.3±0.1	1.0±0.3	3.0±0.4	3.7±1.1	255±98	341±30
PECUU-3	1.2±0.4	1.2±0.4	6.9±0.4	8.0±0.4	519±18	504±39
PECUU-4	0.2±0.1	0.5±0.1	0.6±0.1	0.8±0.4	330±38	450±139

**Table 2:** Mechanical properties of PECUUs after degradation in PBS for different period of time at 37 °C. The samples were prepared by solution cast method (0.2–0.3 mm thickness). Mechanical properties were measured by uniaxial tensile tests (*n*=4).

**Sample**	**Degradation time (week)**	**Young׳s modulus (MPa)**	**Tensile strength (MPa)**	**Elongation at break (%)**
PECUU-1	0	13.4±1.7	8.2±0.8	329±62
1	14.1±1.2	8.3±0.6	205±37
2	12.8±1.4	5.5±0.8	43±8
3	13.0±1.5	3.2±0.5	40±12
PECUU-2	0	6.4±0.5	11.9±1.1	1544±234
1	6.2±0.7	10.4±1.3	1525±126
2	5.5±0.6	5.7±0.9	608±107
3	5.3±0.9	7.0±1.5	973±168
4	5.3±0.5	5.7±1.7	601±112
PECUU-3	0	5.1±0.2	16.1±1.2	2146±88
1	3.7±0.4	7.4±1.5	1096±102
2	3.8±0.6	7.1±1.0	1483±135
3	4.0±0.5	8.0±0.8	1675±134
4	3.7±0.2	5.8±1.2	1155±96
PECUU-4	0	2.5±0.2	2.0±0.2	465±97
1	3.0±0.4	2.6±0.5	806±186
2	2.8±0.5	1.9±0.6	312±79
3	2.7±0.2	2.2±0.3	410±87
4	2.6±0.3	2.0±0.6	412±106

**Table 3:** Summary and side-by-side comparison of the elasticity, biochemistry and traction forces of AFSCs cultured on PECUUs in respect to the corresponding characteristics of native AF tissue.

**Property**		**PECUU scaffold**				**Native AF tissue**		
**PECUU-1**	**PECUU-2**	**PECUU-3**	**PECUU-4**	**Outer**	**Middle**	**Inner**
**Young׳s modulus (MPa)**		13.4±0.7	6.8±0.5	5.1±0.4	2.5±0.4	4.1±0.2	2.1±0.6	0.03±0.002
**Biochemistry**	DNA (μg/scaffold or μg/mg tissue)	8.1±0.2	8.7±0.2	8.8±0.3	6.8±0.2	1.6±0.2	0.9±0.2	0.5±0.1
Collagen I (ng/μg DNA or μg/mg tissue)	22.2±0.6	14.9±0.5	10.4±0.6	5.1±0.5	6.4±0.8	2.6±0.4	1.6±0.4
Collagen II (ng/μg DNA or μg/mg tissue)	2.2± 0.4	6.6±0.2	8.4±0.3	11.2±0.4	0.7±0.2	1.4±0.4	3.9±0.3
GAG (ng/μg DNA or μg/mg tissue)	1.8±0.2	4.5±0.2	5.7±0.2	7.5±0.4	1.5±0.4	7.2±1.4	13.0±1.4
**Cell traction force (Pa)**		251.2±73.5	366.2±67.1	445.3±85.3	559.9±90.1	123.8±76.1	199.0±158.8	336.6±155.3
